# A Panel of MicroRNAs as Diagnostic Biomarkers for the Identification of Prostate Cancer

**DOI:** 10.3390/ijms18061281

**Published:** 2017-06-16

**Authors:** Rhonda Daniel, Qianni Wu, Vernell Williams, Gene Clark, Georgi Guruli, Zendra Zehner

**Affiliations:** 1 Department of Biochemistry and Molecular Biology, VCU Medical Center and the Massey Cancer Center, Virginia Commonwealth University, Richmond, VA 23298-0614, USA; danielr@vcu.edu (R.D.); wuq3@vcu.edu (Q.W.); clarkgc@mymail.vcu.edu (G.C.); 2 Molecular Diagnostic Laboratory, Department of Pathology, VCU Health System, Virginia Commonwealth University, Richmond, VA 23298-0248, USA; Vernell.Williamson@vcuhealth.org; 3 Division of Urology, VCU Medical Center and the Massey Cancer Center, Virginia Commonwealth University, Richmond, VA 23298-0037, USA; Georgi.guruli@vcuhealth.org

**Keywords:** microRNA, high throughput RNA sequencing, small RNA sequencing, qRT-PCR, prostate cancer, PSA

## Abstract

Prostate cancer is the most common non-cutaneous cancer among men; yet, current diagnostic methods are insufficient, and more reliable diagnostic markers need to be developed. One answer that can bridge this gap may lie in microRNAs. These small RNA molecules impact protein expression at the translational level, regulating important cellular pathways, the dysregulation of which can exert tumorigenic effects contributing to cancer. In this study, high throughput sequencing of small RNAs extracted from blood from 28 prostate cancer patients at initial stages of diagnosis and prior to treatment was used to identify microRNAs that could be utilized as diagnostic biomarkers for prostate cancer compared to 12 healthy controls. In addition, a group of four microRNAs (miR-1468-3p, miR-146a-5p, miR-1538 and miR-197-3p) was identified as normalization standards for subsequent qRT-PCR confirmation. qRT-PCR analysis corroborated microRNA sequencing results for the seven top dysregulated microRNAs. The abundance of four microRNAs (miR-127-3p, miR-204-5p, miR-329-3p and miR-487b-3p) was upregulated in blood, whereas the levels of three microRNAs (miR-32-5p, miR-20a-5p and miR-454-3p) were downregulated. Data analysis of the receiver operating curves for these selected microRNAs exhibited a better correlation with prostate cancer than PSA (prostate-specific antigen), the current gold standard for prostate cancer detection. In summary, a panel of seven microRNAs is proposed, many of which have prostate-specific targets, which may represent a significant improvement over current testing methods.

## 1. Introduction

Prostate Cancer (PCa) is the most common non-cutaneous cancer among men, yet current diagnostic methods are insufficient at detecting this disease, and more reliable biomarkers need to be developed. Currently, the prostate-specific antigen (PSA) is used as a diagnostic marker for PCa; however, many factors have been found to elevate PSA levels. Age, infection, trauma, ejaculation, urinary retention, instrumentation, certain medications and even bike riding can lead to false positive diagnoses, generating unnecessary concern and over-treatment with dire outcomes for the patient [1,2,3,4]. Even worse are the chances of false negative diagnoses, which result in PCa remaining undetected until its later stages. Therefore, although the use of the PSA level has had its clinical advantages, it has failed to sufficiently bridge the gap to accurately diagnose disease or distinguish indolent from aggressive disease. One answer that might close this gap and enable more efficient diagnoses may lie in microRNAs (miRs) [5].

Small RNAs play an extremely important role in gene regulation. Their function in the suppression of unwanted genetic materials is vital to the proper operation of the cell. Small RNAs fall into three classifications: microRNAs, siRNA and PIWI-interacting RNAs (piRNA), the most dominating of which are microRNAs [6]. MicroRNAs are small non-coding RNA molecules (18–22 nts in length) that are evolutionarily conserved and associated with the Argonaute family of proteins. These microRNAs function at the translational level through silencing mechanisms to regulate gene expression.

MicroRNAs have been shown to be significantly altered throughout the course of disease progression [7]. This is especially true in cancer where abnormal cell growth and angiogenesis are critical for tumorigenesis to occur. The loss of microRNAs that suppress the translation of oncogenes, termed tumor suppressors, has been shown to contribute to the development and progression of many cancers [7]. These microRNAs are primarily responsible for controlling apoptotic pathways and cell cycle checkpoints [7].

Since the discovery of microRNAs, many research groups have analyzed blood in hopes of establishing a correlation to disease. Mitchell et al. first reported that PCa cells released microRNAs into the bloodstream in protective capsules, the content of which could be monitored by PCR-based methods [8]. Schultz et al. studied whole blood for the identification of microRNAs that could be used as biomarkers for the detection of pancreatic cancer [9]. By confirmatory qRT-PCR, they found 38 microRNAs dysregulated and were able to identify two diagnostic microRNA panels that could distinguish between patients with pancreatic cancer from healthy controls [9]. Another study compared microRNA levels between plasma and serum from PCa patients by measuring four microRNAs: hsa-miR-15b, hsa-miR-16, hsa-miR-19b and hsa-miR-24. Interestingly, they found a strong correlation in the microRNA content of these two types of body fluids supporting either serum or plasma as a sufficient source of material for disease studies [8]. Using qRT-PCR, Cochetti et al. suggested a panel of serum microRNAs that could distinguish PCa from benign prostatic hyperplasia in age-matched patients with elevated PSA levels [10]. Thus, the use of blood, serum or plasma as a worthwhile source of material to diagnose disease is well documented [8,9,10].

To date, a number of studies have used PCR technology to identify microRNAs that could be used as relevant biomarkers to diagnose PCa [11,12]. Certainly, these are important studies, but for the most part, they have used preformed panels of microRNA arrays or focused qRT-PCR assays for specific microRNAs suggested from studying a wide range of different cancers and then applied to PCa. By this approach, only predetermined, known microRNAs are being evaluated. In an effort to widen the scope of microRNA candidates, high throughput sequencing (HTS), also referred to as deep sequencing or RNA sequencing, would better evaluate all possible microRNAs, as well as permitting the discovery of new, novel microRNAs. Keller et al. used HTS of whole blood samples collected with PAXgene blood tubes to study microRNA profiles in lung cancer patients [13]. However, in this case, samples were pooled prior to sequencing, thereby preventing an analysis of microRNA dysregulation across individual samples. To our knowledge, only two reports have used HTS to identify microRNAs diagnostic for PCa. In one case, HTS was used to compare the microRNA content of prostate tumors to adjacent tumor-free margins with the discovery of a loss of miR-143 and miR-145 expression in tumor tissues [14]. A second report applied HTS to exosomal material isolated from blood and found miR-1290 and miR-375 as prognostic markers for castration-resistant prostate cancer (CRPC) [15]. However, in this case, these microRNAs would be useful for identifying late stage prostate cancers.

To better define microRNAs that could be used to more accurately predict PCa at early, not later stages of disease, in this pilot study, we have used HTS of blood from PCa patients at initial stages of diagnosis and before undergoing treatment compared to healthy controls.. Moreover, samples were analyzed individually rather than as a pool so that variability between patients or control samples could be followed. RNA sequencing results were also analyzed to identify normalization microRNAs that could be used as endogenous controls for subsequent qRT-PCR analyses. Confirmatory qRT-PCR was then used to corroborate HTS results for the top seven dysregulated microRNAs. Data analysis of the area under the curve (AUC) of the receiver operating curves (ROC) for these selected microRNAs exhibited a better correlation with prostate cancer (AUC range = 0.819–0.950) than the reported value for PSA (AUC 0.678 comparing PCa to non-cancer) [16]. In summary, a panel of seven microRNAs is proposed, many of which have prostate-specific targets, which upon follow-up confirmatory studies could represent a significant improvement over current testing methods.

## 2. Results

### 2.1. High Throughput Sequencing Results

A summary of the characteristics and pathological data for patients (*n* = 28) and controls (*n* = 12) selected for this study is compared in Table 1. Data for each individual can be found in Appendix Table A1. Blood was retrieved from patients at early stages of diagnosis and prior to treatment. For most cases, age, ethnicity, PSA and Gleason scores obtained from biopsy were reported. The Gleason score was obtained by microscopic analysis by a trained pathologist and is the combined score of the most common and second most abundant cell type based on cell morphology. When the Gleason score or PSA were not available, it is designated as unknown. Although some mix of ethnicity was obtained, Caucasian was most prevalent with no ethnicity or age recorded for nine individuals. Low Gleason scores of G6 and G7 and PSA values ranging from 3.4–22 predominated, since samples were taken from patients at early stages of diagnosis. Although Gleason scores were not reported for four patients, elevated PSA levels including the high of 22 was found within this group supporting their inclusion to analyze as many samples as possible in this pilot study. Every effort was made to select a control group that had no evidence of PCa either for the individual or within the family. PCa being predominately a disease of the elderly, the average age of the patient group did exceed that of the controls, but since all data were analyzed as individuals, we could subsequently evaluate differences within each group. In this case, we did not find notable discrepancies in data within either the patient or control group due to age or the group of four with elevated PSA values, but unknown Gleason scores, further supporting their inclusion in this study.

Blood was collected and small RNAs extracted from individual patient and control samples as described in the Materials and Methods. The HTS data revealed that among the 2588 microRNAs present in the miRBase (mature 21 June 2014) [17], about 550 were found at detectable levels in the samples tested. To better refine this list of potential candidates, *p*-values were adjusted using the Benjamini-Hochberg method to yield a False Detection Rate, or FDR value. The FDR value indicates the possible false detection rate using a generalized linearization model. This method is considered a Type 1 error expansion multiple comparison model that reduces the risk of rejecting a true null hypothesis. In order to include as many positive hits as possible in the HTS screening, a cutoff FDR value of <0.2 was selected. An FDR value of 0.2 would mean that 20% of selected microRNAs may be false positives. Since all HTS results would be subsequently confirmed by qRT-PCR, it was felt that lowering the stringency to include more potential microRNA candidates for future confirmation was acceptable at this initial stage. In fact, lowering the stringency of this selection generated a list of 10 possible dysregulated microRNAs for future study (Table 2). Subsequently, miR-5582-3p and miR-543 were dropped because there were no manufactured primers readily available in the market, and their abundance was low. In addition, miR-500b-3p was also dropped due to its low abundance. Thus, seven microRNAs were chosen for future analysis.

**Figure 1:**
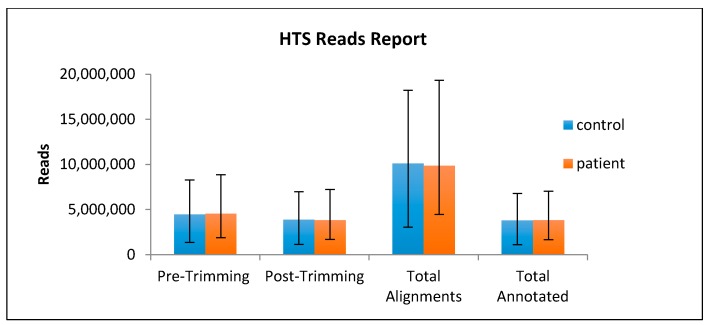
Analysis of HTS reads in blood from PCa patients and controls. HTS was performed on a total number of 40 samples (28 patients and 12 controls) with an RNA concentration of 100 ng/µL. Total reads are shown before and after the Partek Flow^®^ (St. Louis, MO, USA) process.

During the bioinformatics analysis, the HTS total reads for patients and controls were not significantly different from each other, suggesting that blood from normal and patient groups contained similar amounts of total microRNA (Figure 1). This similarity increased the confidence of dysregulation, as it could be confirmed that the differential expression of certain microRNAs was not due to differences in library size.

Processed raw reads were further normalized using the Trimmed Mean of M-values (TMM) method provided by the Edge R program [18]. Based on the hypothesis that most genes are not differentially expressed, the TMM method generates a scaling factor applied to library sizes, which attempts to minimize the intra-group variation in gene expression. This normalization method can further minimize the effect of technical variations caused by sequencing depth and batch variation. The TMM method is a very powerful method when varying library size, and high-count genes can exist [19]. Compared to the commonly-used normalization methods of Total Counts (TC) or Reads Per Kilobase per Million mapped reads (RPKM), the TMM method is more reliable, because it not only normalizes the library size, but also takes into account the effect of RNA composition [18]. The effectiveness of the TMM method in normalizing the microRNA sequencing results was later confirmed via qRT-PCR.

**Figure 2:**
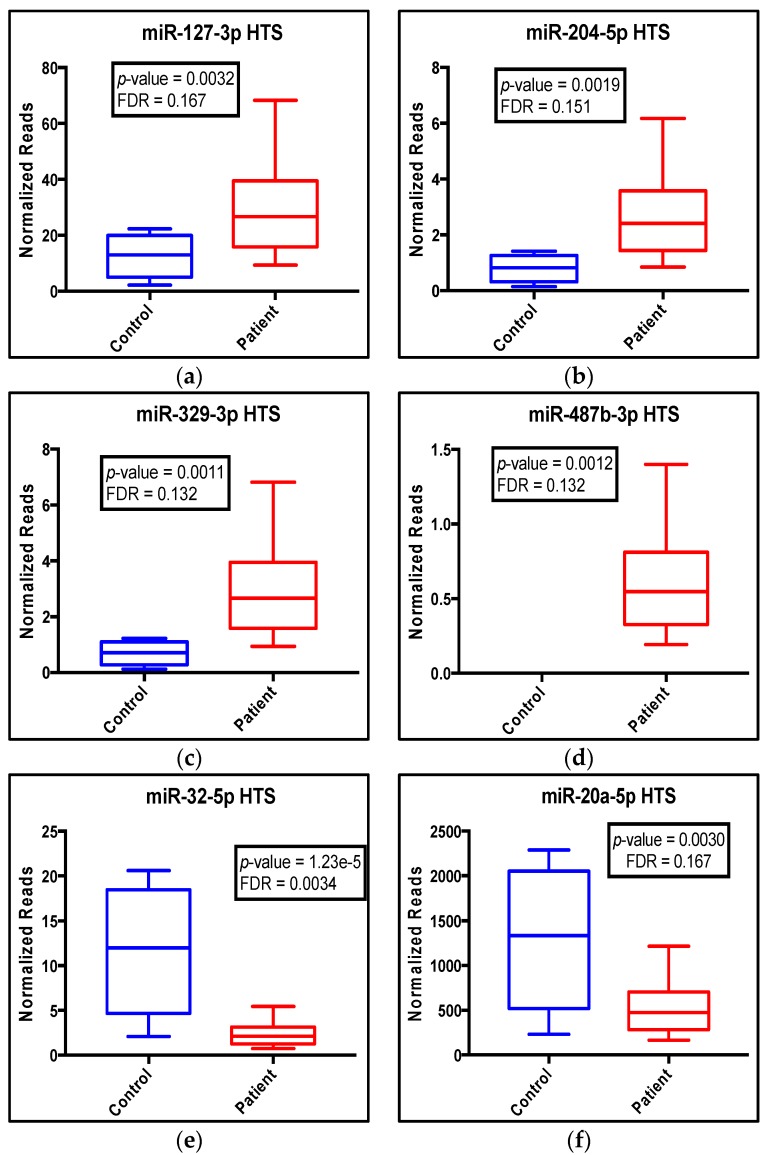

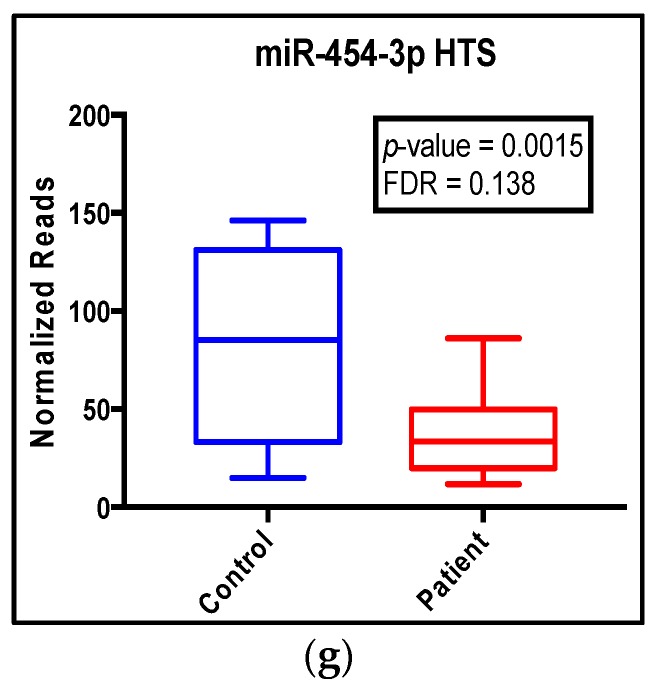
HTS data show dysregulation of seven microRNAs in blood from PCa patients (red) compared to controls (blue). (**a**–**g**) Box plots of the top seven dysregulated microRNAs as indicated on each panel are based on Edge R differential expression analysis. *p*-Values and FDR values were generated by Edge R using the generalized linear method. Box-and-whiskers graphs were plotted using Prism. The minimum, the 25th percentile, the median, the 75th percentile and the maximum are shown on each box plot as the bottom to the top lines, respectively. An FDR < 0.2 was considered significant.

The seven dysregulated microRNAs indicated by HTS differential expression analysis showed great differences in normalized reads between control and patient groups (Figure 2a–g). According to the HTS data, four microRNAs were upregulated (miR-127-3p, miR-204-5p, miR-329-3p and miR-487b-3p) in patients’ blood samples (Figure 2a–d), while three microRNAs (miR-32-5p, miR-20a-5p and miR-454-3p) were downregulated (Figure 2e–g).

### 2.2. Identification of MicroRNAs as Normalization Standards for qRT-PCR

In order to confirm HTS data by qRT-PCR, a normalization method needed to be developed to ensure that microRNA dysregulation was due to true biological variation and not technical error. Ideally, microRNA normalizers should exhibit small standard deviations and display similar expression levels to the dysregulated microRNAs under study. To this end, the concentration of small RNAs in each sample was determined from bioanalyzer results, set to a constant amount, and the *C*q value determined by qRT-PCR. Results were analyzed using the NormFinder program, which scrutinizes intra- and inter-group variations to determine which microRNA candidates are best suited for normalization using an algorithm to calculate a stability value for each microRNA, i.e., the lower the value, the lower the variation.

**Figure 3:**
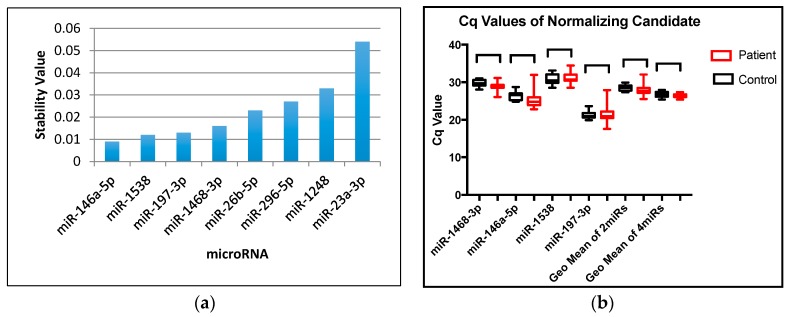
Analysis by the NormFinder program identified four microRNAs as the best normalization candidates for qRT-PCR studies. (**a**) Eight stably-expressed microRNAs (miR-146a-5p, miR-1538, miR-197-3p, miR-1468-3p, miR-26b-5p, miR-296-5p, miR-1248 and miR-23a-3p) suggested by HTS data were confirmed by qRT-PCR in triplicate (eight controls and eight patients). The small RNA concentration for each sample was normalized to roughly 0.012 ng/µL in each reaction. A stability value was generated for each candidate by the NormFinder program, where the lower the value, the better; (**b**) The *C*q value of the top four microRNA candidates (miR-1468-3p, miR-146a-5p, miR-1538, miR-197-3p) was subsequently evaluated in 26 patients and 10 controls and plotted individually as box plots versus the geometric (Geo) mean of two candidates (miR-146a-5p and miR-1538) or four candidates (miR-146a-5p, miR-1538, miR-197-3p and miR-1468-3p ) as analyzed in triplicate by qRT-PCR.

The NormFinder program selected eight microRNAs as exhibiting stable expression patterns; miR-146a-5p, miR-1538, miR-197-3p, miR-1468-3p, miR-26b-5p, miR-296-5p, miR-1248 and miR-23a-3p (Figure 3a; raw data Appendix Table A2). Due to limiting amounts of material, the search for potential microRNA normalizers was initially monitored in a subset of samples, i.e., eight patients and eight controls. Of these, the first four candidates, which exhibited the closest stability values (ranging from 0.009–0.0016) with minimal differences in expression between control and patient samples, were subsequently analyzed in a fuller spectrum of samples (26 patients and 10 controls). Unfortunately, two samples from each group of HTS data had to be dropped from further analysis due to a lack of material (Figure 3b; raw data Appendix Table A3). NormFinder suggested that the single best candidate was miR-146a-5p. However, when there is no obvious single, outstanding normalization candidate, NormFinder suggests using a combination of microRNAs to increase reliability and produce less intra- and inter-group variability. Since the top four microRNAs (miR-146a-5p, miR-1538, miR-197-3p, miR-1468-3p) all showed very close stability values, the *C*q value of each was compared to each other, as well as the geometric mean of the top two candidates (miR-146-5p and miR-1538) or all four top candidates together (Figure 3b). The top two candidates showed decreased intra-group variation, especially for the patient group (Figure 3b). However, the geometric mean of all four candidates together showed even smaller intra- and inter-variations within and between both control and patient groups (Figure 3b). Therefore, these four microRNAs were selected as a group of normalizers to be used for downstream qRT-PCR analyses.

### 2.3. Validation of HTS Data by qRT-PCR Analysis

**Figure 4:**
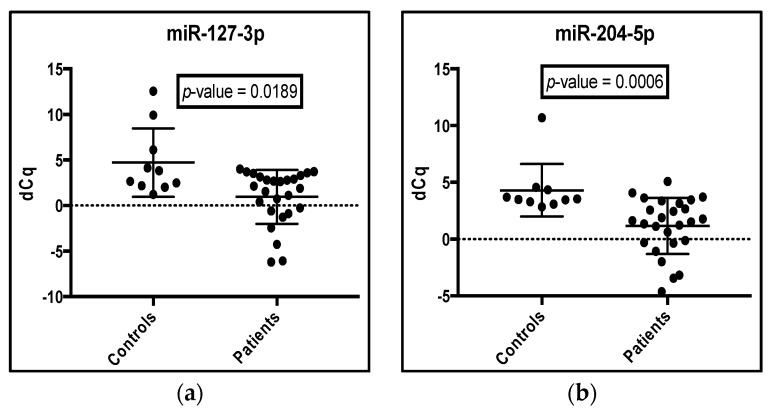

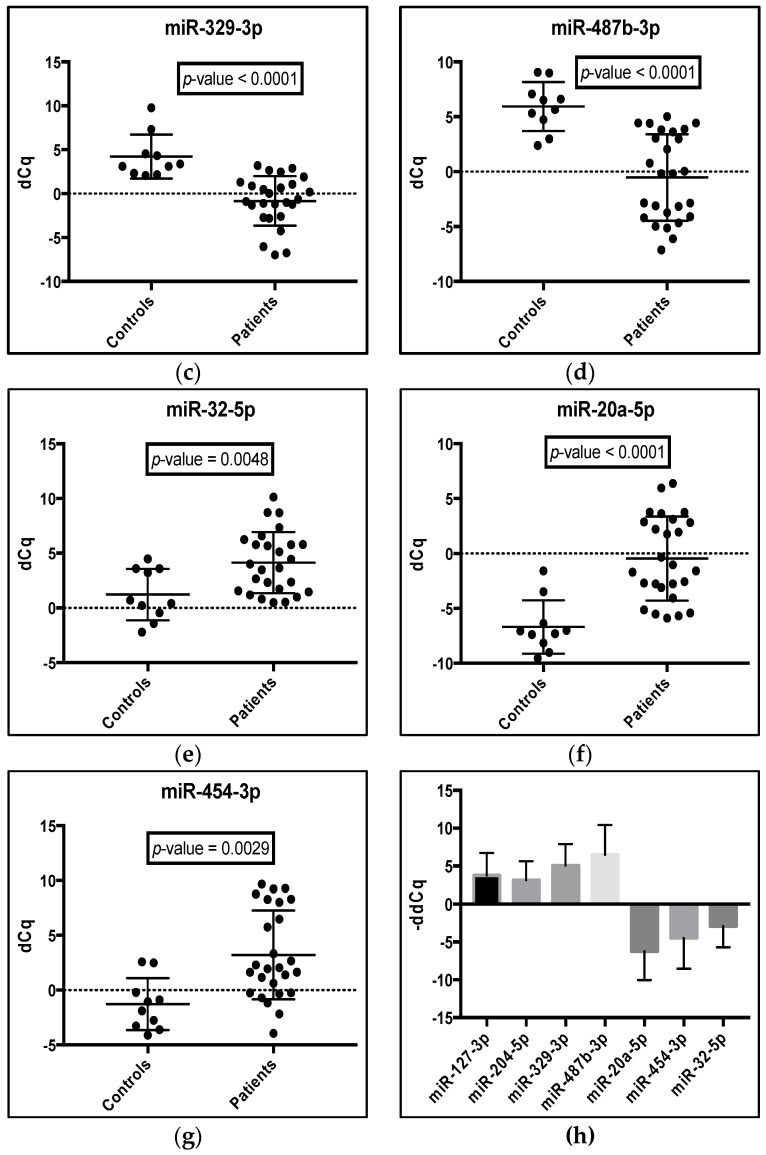
Confirmatory qRT-PCR results for dysregulated miRNA candidates suggested by HTS data. (**a**–**g**) A comparison between normalized *C*q values (d*C*q) from qRT-PCR analysis of blood from patients and controls and plotted as dot blots. qRT-PCR was performed on 36 samples (10 controls and 26 patients) in triplicate. Samples were adjusted to the same small RNA concentration (0.012 ng/µL) per reaction. Raw *C*q values were normalized by subtracting the geometric mean *C*q value of the top four normalization candidates (miR-146a-5p, miR-1538, miR-197-3p and miR-1468-3p) suggested by the NormFinder program from individual *C*q values to generate dCq. A *p*-value was obtained using the Mann–Whitney nonparametric test assuming that data do not follow a Gaussian distribution. A *p*-value < 0.05 was considered significant. The minimum, median and maximum values are shown as respective lines from the bottom to the top; (**h)** The −dd*C*q values of the seven dysregulated microRNAs are shown. The −dd*C*q for each candidate was obtained by taking the mean of the normalized d*C*q of all controls minus the normalized d*C*q of each patient sample. This value equals the fold change on a Log2 scale.

The elucidation of valid microRNA normalizers permitted further analysis of HTS results via qRT-PCR. The individual dot plots of d*C*q (∆*C*q) values are shown for the seven dysregulated miRs suggested by HTS data (Figure 4). According to the qRT-PCR results, miR-127-3p, miR-204-5p, miR-329-3p and miR-487b-3p were all upregulated in patients compared to controls (Figure 4a–d), while miR-32-5p, miR-20a-5p and miR-454-3p were downregulated (Figure 4e–g) The differences in expression in control versus patient samples was calculated for each microRNA as the –dd*C*q (Log2 fold change) and shown in Figure 4h. Raw and normalized *C*q values for controls and patients are included in Appendix Table A3 and Table A4, respectively. Thus, the qRT PCR results agreed with the HTS data, confirming that all seven microRNAs were dysregulated in PCa patients.

**Figure 5:**
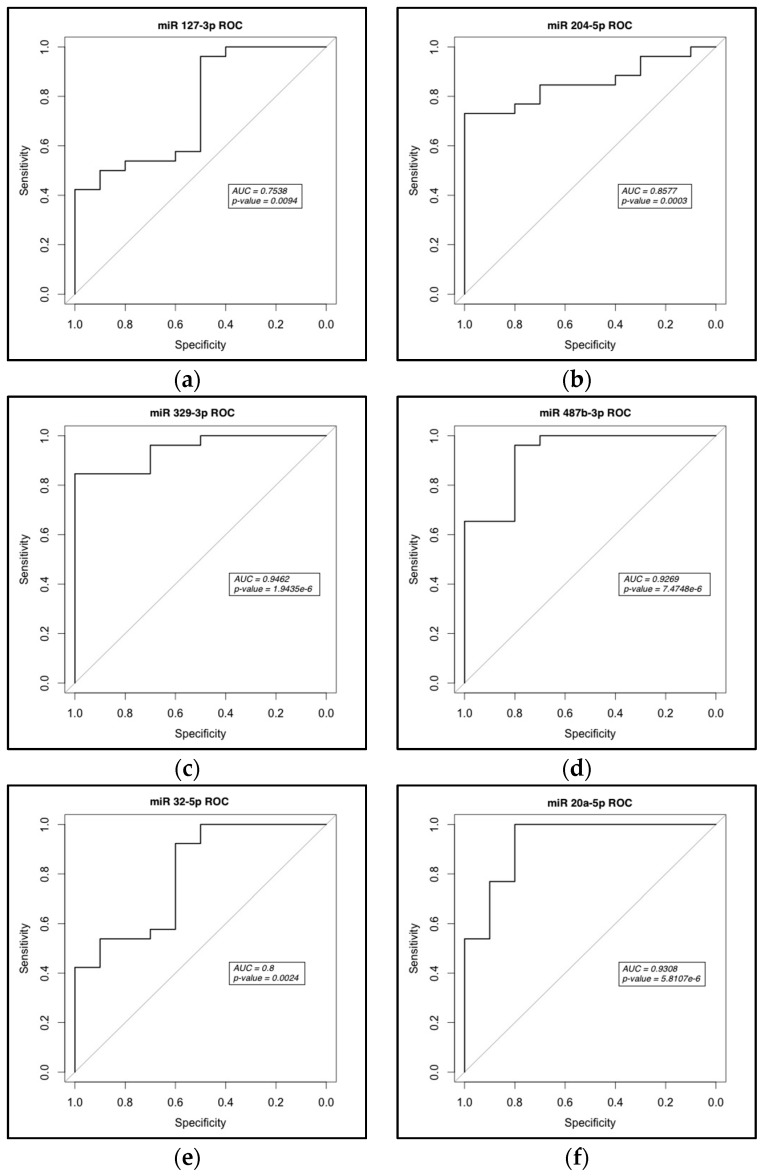

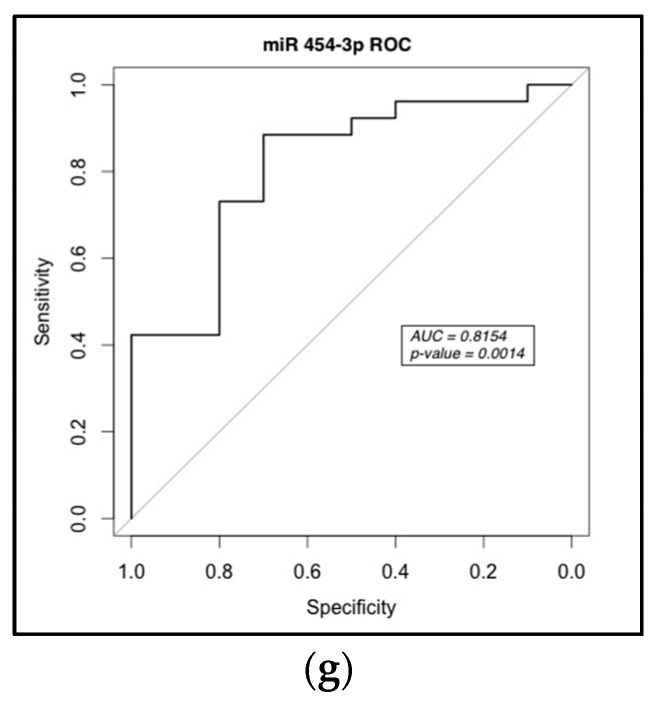
Receiver operator curves for dysregulated microRNAs. (**a**–**g**) Analysis was performed based on the qRT-PCR results in triplicate of individual microRNAs as indicated on each graph and plotted as sensitivity versus specificity. An AUC > 0.5 is considered significant.

In order to further assess whether the seven microRNAs could serve as good biomarkers, Receiver Operator Curves (ROC) were drawn based on the qRT-PCR data. ROC analysis demonstrates the trade-off between sensitivity and specificity where a good biomarker should display both high sensitivity and high specificity [20]. The ROC curve for each microRNA is shown in Figure 5. In ROC analysis, the Area Under the Curve (AUC) quantifies the biomarker potential for each candidate where the higher the AUC value, the better a candidate microRNA is at distinguishing PCa patients from controls. Via ROC analysis, the currently used PCa biomarker, PSA, has a reported AUC value of 0.678 for distinguishing PCa from no cancer [16]. The seven microRNAs identified in our study exhibited a respectable range of AUC values from 0.7538 for miR-127-3p up to 0.9462 for miR-329-3p, all significantly better than that reported for PSA, with *p*-values ranging from 1.9435 × 10^−6^ to 0.0094 (Figure 5a–g).

### 2.4. Comparison of Blood Results to TCGA Database

The expression of our panel of blood microRNAs was compared to expression levels in tumor tissue by our analysis of data in The Cancer Genome Atlas (TCGA) database. Although the TCGA microRNA sequencing data were annotated with the stem-loop transcripts instead of the mature strands, all seven microRNAs from our study derive from the major expressed mature strand of their stem-loop precursor based on data in miRBase [17]. Therefore, the expression of these seven mature microRNAs is directly proportional to the abundance of their stem-loop precursors. The mature miR-127-3p, miR-204-5p, miR-487b-3p, miR-32-5p, miR-20a-5p and miR-454-3p are derived from precursors miR-127, miR-204, miR-487b, miR-32, miR-20a and miR-454, respectively. The mature miR-329-3p was derived from two precursors, miR-329-1 and miR-329-2.

**Figure 6:**
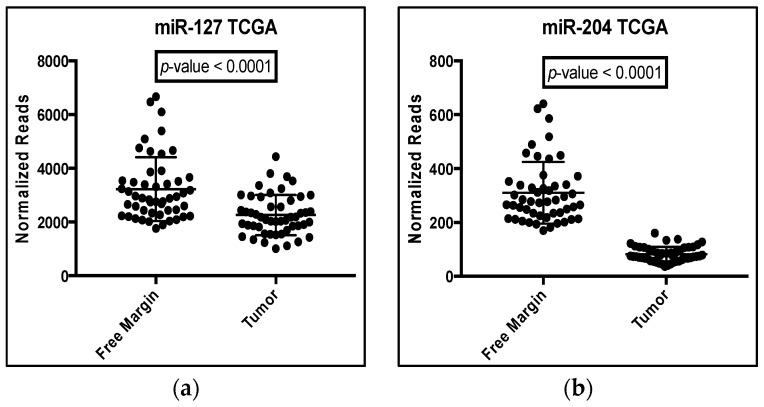

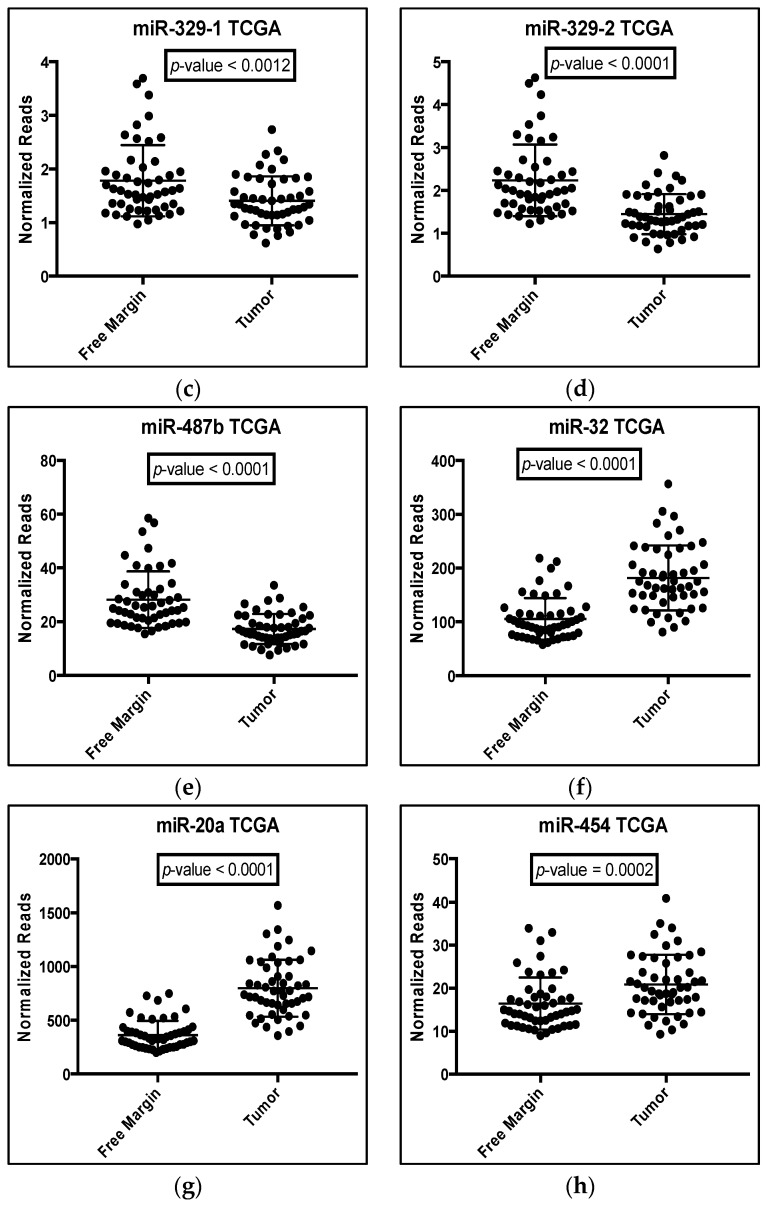
An analysis of microRNA sequencing results from the TGCA matched tissue database for the seven microRNA candidates. (**a**–**h**) Reads for each microRNA candidate as indicated were normalized using the Edge R TMM method and plotted as a dot blot with a line (bottom to top, respectively) representing the minimum, median (or mean) and maximum value for the tumor versus the disease-free matched tissue (free-margin) from PCa patients. A *p*-value was obtained using the Mann–Whitney nonparametric test assuming that that data do not follow a Gaussian distribution. A *p* < 0.05 was considered significant. TCGA, The Cancer Genome Atlas.

The expression of each precursor in PCa tissues compared to their disease-free matched margins showed significant dysregulation, and the direction of dysregulation agreed with the literature results (Figure 6). However, the pattern of dysregulation for each microRNA in tumor tissue was opposite to that pattern observed in our blood samples. For example, miR-127, miR-204, miR-329-1, miR-329-2 and miR-487b were all upregulated in PCa tissue, which suggested that their major mature strands (miR-127-3p, miR-204-5p, miR-329-3p and miR-487b-3p) were also upregulated. However, these four microRNAs were shown to be downregulated in our blood samples. The inverse correlation was observed for the three microRNAs (miR-32-5p, miR-20a-5p and miR-454-3p) that are downregulated in blood. Again, their precursor transcripts and presumably major, mature microRNA products were upregulated in the TCGA tissue data. A comparison of the fold changes between our HTS blood data versus that from the TCGA database are included in the Appendix (Table A5).

## 3. Discussion

Analysis of HTS sequencing results suggested a panel of seven microRNAs that could be useful in diagnosing PCa in blood. Previously, the lack of reliable microRNA standards for normalization across different samples had been detrimental to subsequent qRT-PCR validation studies. In some studies, snRNAs have been used for this purpose, but since these are not normally secreted and are not produced by pathways that correlate with microRNA synthesis, their use as normalizers for complex body fluids such as blood is questionable. A review of our HTS data selected four microRNAs (miR-197-3p or -5p, miR-1538, miR-1468-3p and miR-146a-5p) that were consistently expressed across all patient and control samples and could be used as reliable normalization standards for future qRT-PCR studies. Kirschner et al. also found miR-146a to be stably expressed in plasma and serum, not affected by hemolysis, in agreement with our results in blood [21]. Moreover, the enhanced geometric mean of these microRNAs was shown to be significantly better than any single microRNA alone.

With a proven group of normalization standards, it was important to confirm HTS results via qRT-PCR. Significantly, results from these two very different methodologies agreed well, further supporting the validity of our approach. All of the microRNAs with low *p*- and FDR-values via HTS data showed significant *p*-values with qRT-PCR and notable AUC values upon ROC analysis. This agreement was encouraging because thus far, investigators had been determining diagnostic microRNAs by screening of pre-selected microRNA arrays, which represented only a small subset of microRNAs from the database of >2580 total microRNAs [17]. This approach is limited to analyzing only those microRNAs that have already been shown to be dysregulated in some disease and then selected for analyzing PCa. However, HTS permits the identification of all possible diagnostic miRNAs, both known and perhaps novel, expanding the spectrum of microRNA candidates evaluated. Interestingly, a few novel microRNA species were identified, but these always turned out to be a single report; thus, their relevance as a “new” molecule warranting further verification was hard to justify in this pilot study due to their low abundance. More importantly, HTS results were validated by qRT-PCR for all seven candidates generating ROC curves with individual AUC values better than PSA (AUC = 0.678), the current gold standard for diagnosing PCa [16].

Another value of our suggested panel is that four microRNAs are upregulated in blood, whereas three are downregulated. This result means that each group can serve as an additional internal control for each other, thereby further serving to verify the accuracy of results, i.e., they do not all go up or all go down. Constructing a diagnostic panel with only downregulated microRNAs is always hard to justify; however, by pairing the loss of three microRNAs with an increase in the other four allows for greater diagnostic confidence.

In some previous studies, PCa samples were pooled in order to obtain sufficient material for subsequent analysis [22,23]. This approach prevents an analysis of variability across individual samples and blocks any correlation to the stage of disease when Gleason scores are available. Not only is it important to diagnose PCa, but eventually to identify biomarkers that could serve to stage disease and, more importantly, discern indolent from aggressive disease, thereby impacting subsequent treatment options. Thus, the fact that valid HTS data could be acquired from individual samples without requiring pooling might enable a correlation between microRNA and tumor stage in future studies. Samples analyzed here were predominately from lower Gleason-scored patients (Table 1: 9 or 11 patients with a G6 or G7 score respectively with only 2 samples scored as G8 or G9). Thus, our panel is more diagnostic for early detection and, if these patients could be followed, might elucidate microRNAs useful for separating indolent from more aggressive disease. In any case, more patient samples are needed particularly with higher Gleason scores to determine microRNAs that could identify later stages of disease as proposed for miR-1290 and miR-375 in CRPC [15].

### 3.1. Literature Review of Diagnostic Panel of Dysregulated MicroRNAs in Cancer

A brief review of these diagnostic seven microRNAs, their Chromosomal (Chr) location and known targets was carried out to determine if their dysregulation might support a functional role in prostate tumorigenesis. 

#### 3.1.1. miR-127-3p, miR-204-5p, miR-329-3p and miR-487b-3p as Tumor Suppressors

miR-127-3p (Chr 14) is situated near a cluster of microRNAs (has-miR-431, hsa-miR-433, hsa-miR-432 and hsa-miR-136) susceptible to epigenetic silencing [24]. It has been shown to target BCL6 and is downregulated in breast cancer tissue where overexpression of miR-127-3p or depletion of BCL6 supported its role as a tumor suppressor [25]. In addition, BCL6 plays an important role in cell proliferation by suppressing transcription of the anti-apoptotic *BCL-XL* gene and the adhesion molecule VCAM [26,27].

miR-204-5p (Chr 9) is highly downregulated in many tumor types including breast, kidney and prostate [28]. The absence of miR-204-5p led to a decrease in Kir7.1 proteins, which connect TGF-BR2 and maintain potassium homeostasis, thereby playing a crucial role in maintaining epithelial barrier function and cell physiology [28]. miR-204-5p has been shown to suppress the growth, migration and invasion of endometrial carcinomas by binding to TrkB mRNA and interfering with JAK2 and STAT3 phosphorylation [29].

miR-329-3p (Chr14) is part of an extensive microRNA cluster containing over 40 microRNAs. Yang et al. found miR-329-3p to be downregulated in metastatic, neuroblastoma tumor tissue compared to the primary tumor [30]. One promising target for miR-329-3p is KDM1A, which has been shown to be significantly upregulated in the androgen-dependent LnCaP prostate cell line [30,31]. Upon depletion of KDMA1 using siRNA, VEGF-A expression was also decreased, which in turn blocked androgen-induced VEGF-A, PSA and Tmprss2 expression, suggesting a role for miR-329-3p as a tumor suppressor.

miR-487-3p (Chr 14 within the same microRNA cluster as miR-329) has been found to be downregulated in neuroblastomas and in PCa [32,33]. Moreover, 10 microRNAs from this cluster were found to be significantly downregulated in PCa as Gleason scores increased, thereby playing an important role in regulating proliferation, apoptosis, migration and invasion in metastatic PCa cells [32]. An interesting predicted target for miR-487b-3p is ALDH1A3, aldehyde dehydrogenase 1A3, an enzyme known to be upregulated four-fold in the LnCaP PCa cell lines [34] when exposed to the androgen Dihydrotestosterone (DHT).

#### 3.1.2. miR-32-5p, miR-20a-5p and miR-454-3p as OncomiRs

miR-32-5p (Chr 9) has been found to be an androgen-regulated microRNA that targets BTG2 [35]. Its overexpression has been shown to block apoptosis and promote PCa in CRPC. Furthermore, this microRNA was discovered to be regulated by DHT and displays putative upstream androgen receptor-binding sites (ARBS).

miR-20a-5p (Chr 13) is part of the miR-17–92 cluster, which plays an important role in cell cycle progression, proliferation, apoptosis and other cellular processes [36]. One of the most studied targets of miR-20a is the E2F family, particularly E2F2 and E2F3 [37]. The overexpression of miR-20a-5p in the PC3 PCa cell line was shown to regulate the cell cycle via targeting of E2F2 and E2F3 mRNAs [36]. In addition, this microRNA also targets several cyclin-dependent kinases, including p21 and p57, which halt cell cycle progression. Finally, another notable target is FasI, which promotes cell death [37]. Thus, the major targets of miR-20a-5p promote tumorigenesis and angiogenesis by blocking cell cycle checkpoints [36]. 

miR-454-3p is located on Chr 17 in the first intronic region of its host gene *SKA2* (Spindle and Kinetochore-Associated Complex Subunit 2). SKA2 is essential for proper chromosome segregation. During the cell cycle, both SKA2 and miR-454-3p have been shown to be upregulated. miR-454-3p targets the tumor suppressor gene, *BTG1* (B cell Translocation Gene 1), which plays an important role in cell cycle progression and is involved in the stress response [38]. This anti-proliferative gene is expressed at its highest concentration during the G0/G1 phases of the cell cycle and is then downregulated when the cell progresses through the G1 phase. In renal carcinoma cells, an increase in miR-454-3p displayed a marked decrease in BTG1 via a direct interaction with the 3′-UTR of BTG1 mRNA [38].

#### 3.1.3. Summary of the Literature Review for Targets of Panel MicroRNAs

A summary of these results is shown in Table 3. The four upregulated microRNAs in patient blood (miR-127-3p, miR-329-3p, miR-487b-3p and miR-204-5p) cumulatively target BCL6, TrkB, KDM1A and ALDH1A3, all of which have been shown to be important regulators in PCa [24,25,26,27,28,29,30,31,32,33,34]. Since these proteins exert oncogenic effects in prostate tissue, their regulators are viewed as tumor suppressors, the loss of which could contribute to tumorigenesis. On the other hand, the three downregulated microRNAs in patient blood (miR-20a-5p, miR-32-5p and miR-454-3p) have been shown to target the tumor suppressor proteins E2F2/3, BTG2 and BTG1, respectively [35,36,37,38]. Although they were downregulated in our patient blood samples, they have been shown to be oncomiRs in tumor tissue, the retention of which could promote tumor progression. A review of this literature supports how these microRNAs could play a role in PCa progression.

Interestingly, three of the microRNAs in our panel belong to the same mega cluster on Chr 14. A post-review of our data did note differential expression for several additional members from this cluster. However, due to their low abundance, slightly higher FDR values and limited budget, they were not included in subsequent qRT-PCR confirmatory studies. Analysis of the HTS data showed that five mega cluster members (miR-654-5p, miR 654-3p, miR-493-3p, miR-493-5p and 433-5p) were present in the top 50 dysregulated microRNAs ranking 17th–59th from the top (Appendix Table A6). A review of the TGCA data showed that all were downregulated to different degrees in tumor tissue, fitting with their loss as tumor suppressors. Interestingly, one of these microRNAs, miR-433-3p, has been shown to target CREB (cAMP Response Element Binding protein), a nuclear transcription factor shown to be involved in tumor initiation, progression and metastasis [39]. Sun et al. showed that overexpression of miR-433-3p could counteract the effects of CREB. Studies have shown that the microRNAs in this Chr 14 cluster are downregulated through unknown mechanisms. If increases in these microRNAs are found in blood, it is possible to hypothesize that the expression of these microRNAs is not just being turned off at the transcriptional level, but that they are being shuttled out of the tumor cell and into the blood as a survival and growth mechanism for the developing tumor.

### 3.2. Relevance of Comparing Blood HTS and qRT-PCR Data to the TCGA Database

**Figure 7:**
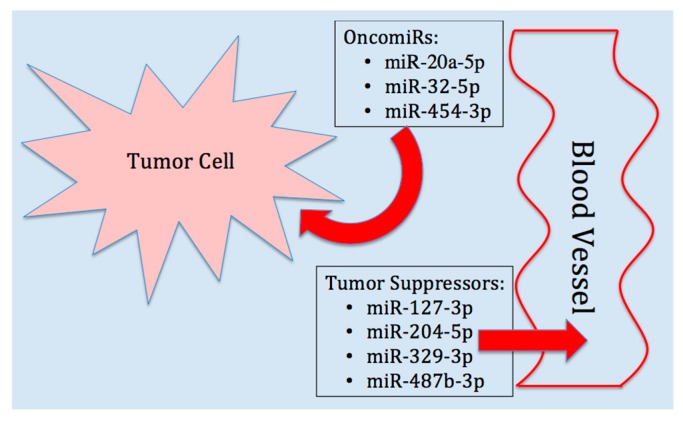
Relationship between microRNA content in prostate tumor cell derived from the TCGA database versus our analysis of blood in prostate cancer patients. The tumor cell retains oncomiRs, but disposes of tumor suppressors to enhance tumorigenesis.

An unanticipated discovery from this study was the inverse relationship between blood and tumor microRNA expression levels (Figure 2 and Figure 4 compared to Figure 6). Since all of our blood microRNAs displayed an inverse expression level with our analysis of data from the TCGA database, we propose that it is possible that tumors are retaining oncomiRs for the purpose of driving tumorigenesis and angiogenesis, and therefore, less of these oncomiRs are released into the blood (Figure 7). Conversely, tumor suppressors block tumor growth and may need to be disposed of to enhance tumorigenesis and ultimately metastasis; hence, the increase in blood levels of these microRNAs. If this were the case for only one or two members of our diagnostic panel of seven microRNAs, perhaps not, but the recurrence for all seven candidates lends credibility to this hypothesis. Moreover, a review of their known targets and the roles they could play in PCa further supports this idea (Table 3).

It has been shown that cancer cells secrete vesicles containing not only mature microRNAs to modify their environment for future metastasis, but the entire processing machinery (dicer, RISC with premiR) to ensure that once taken up by the target cell, the microRNA is efficiently processed and actively moved into the translational silencing mechanism of target mRNAs [40]. It is proposed that if only the mature microRNA were delivered to a secondary site, it might not be as efficient in modifying translation within the target cell. Thus, the preferential cellular export of certain microRNAs as “hormomirs” may function to modulate gene expression at secondary sites, thereby affecting disease pathology [41,42]. With this in mind, it is not much of a stretch to propose a developing tumor wants to dispose of compromising microRNAs that could restrict its growth; thus, excluding tumor suppressor microRNAs. Concomitantly, holding onto an oncomiR to quickly modulate the proteome is fast and efficient, faster than modifying gene expression at the transcriptional level. In support of this hypothesis, Selth et al. found a similar inverse relationship between the loss of miR-146b-3p expression in the prostate tumor with a concomitant increase in circulation [41]. Conversely, in the same study, a direct correlation between increased expression of miR-194 in the tumor and in circulation was noted, suggesting that microRNAs may vary in their expression and patterns of secretion. Since overexpression of miR-194 blocks cell proliferation, induces apoptosis, caspase-3/-9 activities and p53/p21 signaling while suppressing PI3K/AKT/FoxO3a signaling, it is difficult to understand how a tumor could tolerate an increase in the expression of this microRNA, which was not discussed [43]. Another study found miR-194 to be decreased in prostate tumors, befitting its function as a tumor suppressor [44]. On the other hand, miR-1 and miR-133a have been shown to be increased in serum in response to acute myocardial infarction where the levels of both are reduced in the infarcted myocardial tissue [45]. Thus, some evidence for an inverse correlation between tissue expression and microRNAs in circulation exists, but at this time, additional studies in PCa with more patient samples and expansion to other cancers and disease states need to be completed to determine the overall merits of this hypothesis. 

### 3.3. Comparison of HTS Data to Screens of MicroRNA Panels and qRT-PCR Analysis

To date, the elucidation of microRNAs to identify PCa has been mostly generated from screens of preformed microRNA panels or qRT-PCR assays for microRNAs already shown to be involved in cancer. In both cases, the decision as to what microRNAs should be surveyed has already been made rather than using a technology like HTS, which uses no preselection and permits the identification of any potential microRNA. Cochetti et al. chose 23 microRNAs from an in silico survey of predicted target genes to analyze serum from PCa patients [10]. A review of a variety of such studies using serum or plasma has suggested miR-141, -21, -200b, -375, -221, -26a, -195, -15b, -16, -19b, -24, -451 or let-7i as biomarkers to distinguish PCa patients from healthy individuals [5,8,14,41,42]. Some of these microRNAs have also been proposed as biomarkers for other cancers not being unique to PCa (miR-141, -21, -16, -451) or are heavily influenced by hemolysis (miR-15b, -16, -451), making their utility for PCa diagnosis debatable [8,42]. Interestingly, we did not find any of these microRNAs to be significantly altered in our HTS data. In part, this could be due to differences in analyzing serum or plasma versus blood, a very different source of body fluid, as well as the use of HTS data as the starting point for investigation.

HTS has been applied to a very limited number of studies for identifying microRNAs diagnostic for PCa. Szczyrba et al. looked at microRNA profiles of prostate carcinoma compared to normal tissue and cell lines [14]. Here, the loss of miR-143 and miR-145 targeting myosin VI (MYO6) was suggested as a diagnostic marker for prostate carcinoma. However, we did not find these two microRNAs to be dysregulated in blood. Of more significance to our study was the reported HTS data of blood exosomal material isolated from CRPC patients [15]. Here, as well, a group of normalizing RNAs (miR-301a/e-5p, miR-99a-5p, let-7c, miR-125a-5p, miR-16-5 and RNU6B) was proposed for subsequent qRT-PCR analysis. Since RNU6B is not a secreted microRNA, it is doubtful that it should have been included in this analysis, but discounting this snRNA, the rest would be useful normalizers for exosomal material. Interestingly, we found a completely different group of normalizing microRNAs with no overlap of this group, supporting how different an exosomal pool might be to the blood samples analyzed here. Upon normalization, these authors proposed miR-1290 and miR-375 as prognostic markers for CRPC. Since this was only for CRPC patients, perhaps it is not surprising that we did not find these two microRNAs in our analysis, since we are not focused on CRPC. Perhaps our panel of seven microRNAs would be better suited for identifying early stages of prostate cancer, rather than this later stage. Again, additional studies for CRPC versus patients that have not progressed to later stage disease are needed to clarify this hypothesis.

### 3.4. Limitations to This Pilot Study

This study was meant as a pilot study and, as such, suffers from some limitations, which should be addressed. First, to obtain sufficient material for HTS analysis, blood was used as the initial source of small RNAs. Thus, small RNAs will be contaminated with cellular microRNAs, not just circulating microRNAs. However, circulating microRNAs can come in many forms as exosomes, microvesicles, apoptotic bodies or bound to HDL, argonaute 2 or RNA-binding proteins, such as nucleophosmin 1 [42]. At this time, it is not clear which of these forms or combinations thereof would be the most diagnostic as biomarkers for PCa. Thus, focusing on some particles (exosomes or microvesicles) at the exclusion of the others might not be the most relevant source. Isolation of small RNAs from whole blood rather than purification of a subset of these particles seemed like a more inclusive starting point. More importantly, it was assumed that control samples will contain the same contaminates as patient samples, and thus, contaminating cellular microRNAs should cancel out and not be found amongst the dysregulated microRNAs, if samples are handled consistently. In support of this premise, it was reassuring that microRNAs known to reflect WBC (miRs-15- and -230), RBCs affected by hemolysis (miR-16-485-3p, -532-3p, -15b, -16 and -451), RBCs not-affected by hemolysis (miR-1274b, -142-3p and 146a), myeloid (miR-7a, -223, -197 and 574-3p) or lymphoid (miR-150) cells were not found in our panel of dysregulated microRNAs [21,41,42,46]. Our final panel of seven microRNAs was unique amongst those proposed in the literature, perhaps due to the fact that they were compiled from HTS data rather than screens of predetermined miR array panels or primer sets. Second, PSA values were not reported for control samples, making it impossible to obtain an AUC value for this cohort. Thus, an AUC value for PSA was taken from the literature [16], and it could be higher for our control group with its younger age, a problem encountered by other such studies [42]. However, all seven panel members yielded individual AUC values considerably better than the reported value for PSA, which when used as a panel should be stronger than any single microRNA. In fact, in a review by Selth, it was proposed that “no single analyte is likely to achieve the desired level of diagnostic or prognostic accuracy for PCa … requiring a signature of multiple microRNAs rather than a single miR”, as proposed here [42]. Third, validation of HTS results by qRT-PCR was on the same cohort of samples analyzed by HTS. In a future study, a third larger cohort should be evaluated independently to better validate panel members as relevant biomarkers. Finally, these samples did not span the spectrum of higher Gleason scores, but reflect earlier stages of PCa. Thus, at this time, they are not useful for staging or separating indolent from aggressive disease, an important future correlate. Nevertheless, it is proposed that despite these limitations, this initial pilot study does present new novel microRNAs that have not been previously suggested, which warrant inclusion in future studies sampling larger cohorts.

Here, we have proposed a panel of seven microRNAs generated from HTS data rather than pre-judged screens of microRNA arrays proposed from other cancers with unknown relevance to PCa. The same criticism exists for data generated by qRT-PCR studies, since again, only a subset of chosen total microRNAs is being investigated. We propose that HTS data confirmed by qRT-PCR analysis are a worthwhile approach for deducing biomarkers for PCa. Certainly, our ROC curves and AUC values appear superior compared to the current PSA gold standard [16]. As a group, the value of a panel of diagnostic microRNAs is substantial. However, additional studies with more extensive patient sampling are required to determine their future usefulness in not only identifying PCa, but ultimately staging prostate cancer and separating indolent versus aggressive disease. This will require sampling, preferably individually, of a vast number of patient and control samples, but our initial study certainly justifies the merits of future investigation.

## 4. Materials and Methods

### 4.1. Sample Extraction and HTS Sequencing

Whole blood samples from patients and controls were obtained from the Nelson Urology Clinic, VCU (Virginia Commonwealth University) Medical Center and Mcguire Veterans Hospital following approval by the ethics committee (IRB Panel D Approval #HM14344). All patients provided written consent. Blood samples were taken prior to treatment, radiotherapy or prostatectomy. In most cases, age, ethnicity, PSA values and Gleason scores from biopsies were provided (Table 1). Controls were carefully selected to not have any history of PCa either as an individual or within the family, and written consent was obtained. A complete analysis of information provided to us for each individual is included in Appendix Table A1. Samples were collected in PAXgene blood tubes (PreAnalytiX, Qiagen/BD, Franklin Lakes, NJ, USA), which contain a manufacture’s additive to stabilize RNA. The total RNA, including microRNAs in each sample, was extracted using a corresponding PAXgene blood miRNA kit following the manufacture’s protocol, which removes DNA and results in the purification of pure RNA. The quality and concentration of small RNAs ranging from 10–40 nts were measured using the small RNA Chip Assay (Agilent) based on the manufacturer’s instructions. A total number of 40 samples (12 controls and 28 patients) with a small RNA concentration >100 ng/µL was selected for HTS microRNA sequencing using the Illumina^®^ TruSeq Small RNA Library Preparation kit (New England Biolabs, Ipswich, MA, USA) and HiSeq 2500 system (Illumina, San Diego CA, USA) according to the manufacturer’s protocol.

### 4.2. Bioinformatics Analysis

The raw deep sequencing data were processed using Flow^®^ v 3.0 (Partek Incorporated, St. Louis, MO, USA). The adapter sequence “AGATCGGAAGAGCACACGTCT” (TruSeq Adapter, Index 7), frequently detected from all reads, was removed from both the 5′- and 3′-ends. A second trimming was performed to further eliminate bases at both ends with a Phred quality score lower than the average 35, indicating a probability that every 1 in 5000 bases was incorrect; accuracy of 99.95%. The minimum read length detected by the program was changed from 25 to 16 nts in order to include all possible microRNA reads in a suitable range. The trimmed data were aligned to the human genome (GRCh38) with only 1 seed mismatch allowed. The three best alignments satisfying such criteria were reported for each read using Bowtie 1.0. The parameters used by Bowtie while performing the alignment were as follows: alignment mod = quality limit, seed mismatch limit = 1, seed length = 28 and quality limit = 70, both strand alignment and alignments reported per read = 3. The aligned reads were annotated with miRBase (mature 21, Version 2). The differential expression analysis was conducted on the annotated sequencing reads exported from Partek Flow^®^ using Edge R (Version 3.12) (Roswell Park Cancer Institute, Buffalo, NY, USA) [18,47]. The reads were normalized using the default “Trimmed Mean of M values” (TMM method) algorithm, which aims at minimizing the effect of sequencing depth and RNA composition [47].

### 4.3. Quantitative Real Time-Polymerase Chain Reaction

The search for microRNA candidates serving as good qRT-PCR endogenous controls was attempted using NormFinder software [48]. The microRNAs showing a relative high abundance and minimal intergroup variation suggested by the HTS data were selected for exploratory qRT-PCR on 16 samples (8 controls, 8 patients) in triplicate (Appendix Table A2). Each RNA sample (3 ngs) was converted to cDNA (final volume 20 µL) using the qScript™ synthesis kit (Quanta Biosciences Inc., Gaithersburg, MD, USA) following the manufacturer’s protocol and qPCR conducted as previously described [49]. Briefly, the cDNA was diluted with RNase-free water 1:1 (*v*/*v*), and 2 µL were used for each PCR reaction run in triplicate. Each PCR reaction was scaled down to 6.25 µL SYBR^®^ Green Master Mix, 0.25 μL primer, 4.0 µL RNase-free H_2_O for the purpose of saving reagents without compromising the results. qRT-PCR was conducted in an Applied Biosystems 7500 real-time PCR instrument (Life Technologies, Foster City, CA, USA) using the following conditions: 50 °C for 2 min, followed by 40 cycles at 95 °C for 15 s, 60 °C for 15 s and 70 °C for 30 s. Data were collected at 70 °C and analyzed using SDS software v1.3.1 (Life Technologies), using automatic threshold and baseline settings. Negative amplification controls and DNase-treated controls were routinely included for each microRNA and did not impact analysis. PCR efficiency for each microRNA primer set was tested and found to be within the acceptable range (80–110%). The *C*q values of each candidate microRNA were imported into the NormFinder program, which generated stability values for each candidate after evaluating both intra- and inter-group variations. Lower stability values suggested higher consistency of a microRNA across different samples and groups. A combination of the four microRNAs with the lowest stability values showed a greater stability and consistency. Therefore, a normalization factor for each plate was determined by taking the geometric mean of the *C*q values of the four microRNAs.

Next qRT-PCR was performed on dysregulated candidate microRNAs using 10 controls and 26 patients (raw and normalized values in Appendix Table A3 and Table A4, respectively) analyzed in triplicate (SD < 0.2). Unfortunately, material from two patient and control samples evaluated by HTS was found to be insufficient for confirmatory qRT-PCR analysis. For all other samples, the protocol was the same as described above. Raw *C*q values were normalized by subtracting the geometric mean *C*q value of the top four normalization candidates (miR-146a-5p, miR-1538, miR-197-3p and miR-1468-3p) from individual *C*q values to generate dCq. A *p*-value was obtained using the Mann–Whitney nonparametric test assuming that data do not follow a Gaussian distribution. A *p*-value <0.05 was considered significant. The −dd*C*q value for each candidate was obtained by taking the mean of the normalized d*C*q for all controls minus the normalized d*C*q of each patient sample. The −dd*C*q values were equivalent to Log2 fold change, as the fold change was calculated by 2^−ddCq^. 

### 4.4. The Cancer Genome Atlas Tissue Data Analysis

Illumina HiSeq level 3 miRNA sequencing data of 50 prostate tumor tissue samples and their matched normal margins were selected and downloaded from The Cancer Genome Atlas database (TCGA database) for analysis. The reads were annotated with stem-loop transcripts of each microRNA [50]. The raw reads were normalized in the same way as described above using the Edge R software [18].

### 4.5. Statistical Analysis

Differential expression analysis was conducted on the normalized next generation sequencing reads for both blood samples and TCGA tissue data using EdgeR [18]. As the distribution of microRNA sequencing reads remains unclear, the dispersion of reads was estimated via the Cox–Reid profile adjusted likelihood method default in Edge R [51]. The reads matrix was fitted to a generalized linear model and a likelihood ratio test was performed on the fitted data. The *p*-value was adjusted to the number of comparisons (equal to total number of microRNAs detected in the sequencing) using the Benjamini–Hochberg method, which yields a False Discovery Rate (FDR) to minimize Type I error [52]. In order to maximize the screening results, a FDR value smaller than 0.2 was considered significant in this experiment. 

For dysregulated microRNA PCR results, the normalized d*C*q values of each candidate between control and patient groups were compared using the Mann–Whitney nonparametric test assuming that the data do not follow a Gaussian distribution on Prism (GraphPad Software Inc., Version 6, 2015). A *p*-value lower than 0.05 was considered significant. ROC curves were generated for candidate microRNAs showing a statistically-significant difference between two groups [20]. The ROC was obtained by plotting sensitivity against specificity using the pROC package (Version 1.8). An area greater than 0.5 under the curve (AUC) suggests the diagnostic potential of each microRNA candidate.

## 5. Conclusions

In summary, we propose a group of four microRNAs (miR-146a-5p, miR-1538, miR197-3p and miR-1468-5p) that could be used as normalization standards for the comparative analysis of blood samples at least for PCa and perhaps other cancers, as well. In addition, a panel of seven microRNAs (miR-127-3p, miR-204-5p, miR-329-3p, miR-487b-3p, miR-32-5p, miR-20a-5p and miR-454-3p) might be useful for diagnosing PCa dependent on further validation. Individual members of this panel display better diagnostic capabilities than PSA alone and as a group are superior.

## Figures and Tables

**Table 1:** Characteristics and pathological data of patients and controls involved in the study.

Characteristics	PCa (*n* = 28)	Controls (*n* = 12)
AGE (years)		
Range Age	55–92 (*n* = 19)	23–91 (*n* = 12)
Mean Age	65.9	50
Unknown	9	0
ETHINICITY (race)		
Caucasian	12	9
African American	6	1
Asian/Hawaiian	1	1
Unknown	9	0
PSA (Prostate Specificity Antigen)		
Range	3.2–22 (*n* = 19)	--
Mean	7.39	--
Elevated	*n* = 3	--
Unknown	*n* = 6	--
PATHOLOGY (Gleason Score)		
G6	*n* = 9	--
G7	*n* = 11	--
G8	*n* = 2	--
G9	*n* = 2	--
Unknown	*n* = 4	--

**Table 2:** HTS differential expression analysis the top 10 dysregulated miRNA candidates.

MicroRNA	LogCPM	LogFC	*p*-Value	FDR
miR-5582-3p	0.861	−2.477	2.36 × 10^−6^	0.001
miR-32-5p	2.436	−2.036	1.23 × 10^−5^	0.003
miR-500b-3p	1.141	2.035	5.77 × 10^−4^	0.105
miR-329-3p	1.760	2.096	1.11 × 10^−3^	0.132
miR-487b-3p	0.854	2.596	1.20 × 10^−3^	0.132
miR-454-3p	5.239	−0.933	1.50 × 10^−3^	0.138
miR-204-5p	1.646	1.781	1.93 × 10^−3^	0.151
miR-20a-5p	9.049	−1.085	2.97 × 10^−3^	0.167
miR-127-3p	4.337	1.433	3.16 × 10^−3^	0.167
miR-543	3.353	1.359	3.48 × 10^−3^	0.167

LogFC = Log2 comparing patients to controls. FDR = false detection rate.

**Table 3:** Summary of targets and their role in prostate cancer for the miRNA panel.

MicroRNA	Validated Target	Possible Role in Cancer
miR-127-3p	BCL6 [24,25,26,27]	Tumor suppressor
miR-204-5p	TrkB [28,29]	Tumor suppressor
miR-329-3p	KDMA1 [30,31]	Tumor suppressor
miR-487b-3p	ALDH1A3 [32,33,34] *	Tumor suppressor
miR-32-5p	BTG2 [35]	OncomiR
miR-20a-5p	E2F family, P21, p57 [36,37]	OncomiR
miR-454-3p	BTG1 [38]	OncomiR

* A predicted target.

## Abbreviations

ALDH1A3	Aldehyde Dehydrogenase 1A3
ARBS	Androgen Receptor-Binging Sites
AUC	Area Under the Curve
BCL6	B-Cell Lymphoma 6 Protein
BCL-XL	B-Cell Lymphoma-Extra Large
BTG1	B Cell Translocation Gene 1
Chr	Chromosome
CPM	Counts Per Million
CREB	Cyclic-AMP Response Element Binding Protein
CRPC	Castration-Resistant Prostate Cancer
Cq	Quantification Cycle
FC	Fold Change
FDR	False Detection Rate
hsa	A Human microRNA
HTS	High Throughput Sequencing
JAK2	Janus Kinase 2
KDM1A	Lysine Demethylase 1A
LnCaP	Androgen-Sensitive Human Prostate Adenocarcinoma Cells derived from the left supraclavicular lymph node metastasis from a 50-year old caucasian male
miR	MicroRNA
PIWI	P-Element-Induced Wimpy Testis
PSA	Prostate-Stimulating Antigen
PCa	Cancer of the Prostate
qRT-PCR	Quantitative Reverse Transcription Polymerase Chain Reaction
RPKM	Reads Per Kilobase of transcript per Million mapped reads
SKA2	Spindle and Kinetochore-Associated Complex Subunit 2
siRNA	Small interfering RNA
snRNA	Small nuclear RNA
STAT3	Signal Transducer and Activator of Transcription 3
TGF-BR2	Transforming Growth Factor Beta Receptor, Type II
TC	Total Counts
TCGA	The Cancer Genome Atlas
TMM	Trim Mean of M values
Tmprss2	Transmembrane protease, serine 2
TRKB	Tropomyosin Receptor Kinase B
UTR	Untranslated Region
VEGF-A	Vascular Endothelial Growth Factor A
VCAM	Vascular Cell Adhesion Protein

## Appendix A

**Table A1:** Characteristics and pathological data of individual patients and controls involved in the study.

Sample Name	Sample Type	Gleason Score	Age	PSA	Race/Ethnicity	HTS Sample	Confirmatory PCR Sample	NormFinder Sample	Number in Appendix
Z1B-SEQ 1	Patient	G9	92	--	Caucasian	yes	yes		Sample 36
Z2B-SEQ 1	Patient	G7	--	--	--	yes	yes		Sample 26
Z3B-SEQ 1	Patient	G7	--	--	--	yes	yes		Sample 27
Z4B-SEQ 1	Patient	G7	--	--	--	yes	yes		Sample 28
Z5B-SEQ 1	Patient	G9	--	--	--	yes	yes		Sample 35
Z6B-SEQ 1	Patient	--	--	Elevated	--	yes	yes		Sample 11
Z7B-SEQ 1	Patient	--	--	22		yes	yes		sample 12
Z8B-SEQ 1	Patient	--	--	Elevated		yes	yes		Sample 13
091714-SEQ 2	Control	Control	51	--	Caucasian	yes	no		--
Case100-SEQ 2	Patient	G7	61	7.78	African American	yes	yes	yes	Sample 24
10212014FAM-SEQ 2	Control	Control	62	--	--	yes	yes	yes	Sample 10
12172014a-SEQ 2	Patient	G7	--	--	--	yes	no		--
12172014b-SEQ 2	Patient	--	--	Elevated	--	yes	no		--
12192014-SEQ 2	Control	Control	51	--	Caucasian	yes	yes	yes	Sample 8
Case18-SEQ 2	Patient	G6	68	6.8	African American	yes	yes	yes	Sample 14
Case20-SEQ 2	Patient	G6	64	4.15	African American	yes	yes		Sample 18
Case31-SEQ 2	Patient	G6	67	12.47	Caucasian	yes	yes	yes	Sample 15
Case33-SEQ 2	Patient	G6	63	3.2	Caucasian	yes	yes		Sample 21
Case36-SEQ 2	Patient	G6	55	10.73	Caucasian	yes	yes	yes	Sample 22
Case40-SEQ 2	Patient	G7	66	3.42	Caucasian	yes	yes		Sample 29
Case56-SEQ 2	Patient	G7	68	4.57	Caucasian	yes	yes		Sample 30
Case72-SEQ 2	Patient	G8	63	4.77	Caucasian	yes	yes		Sample 34
Case85-SEQ 2	Patient	G7	65	6.1	Caucasian	yes	yes	yes	Sample 23
Case9-SEQ 2	Patient	G6	55	8.82	Asian/Hawaiian	yes	yes		Sample 20
Z9B-SEQ 2	Control	Control	54	--	Caucasian	yes	yes	yes	Sample 7
Z10B-SEQ 2	Control	Control	39	--	African American	yes	yes		Sample 6
03242015-SEQ 3	Control	Control	46	--	Caucasian	yes	yes	yes	Sample 4
03262015-SEQ 3	Control	Control	43	--	Caucasian	yes	yes	yes	Sample 3
04062015-SEQ 3	Control	Control	91	--	Caucasian	yes	yes	yes	Sample 9
04242015-SEQ 3	Control	Control	59	--	Caucasian	yes	yes	yes	Sample 5
04282015-SEQ 3	Control	Control	55	--	Asian/Hawaiian	yes	no		--
Case16-SEQ 3	Patient	G8	76	7.99	Caucasian	yes	yes	yes	Sample 33
Case51-SEQ 3	Patient	G7	64	6.06	Caucasian	yes	yes	yes	Sample 25
Case 6-SEQ 3	Patient	G6	58	11	African American	yes	yes	yes	Sample 16
Case82-SEQ 3	Patient	G7	62	8.36	African American	yes	yes		Sample 31
Case83-SEQ 3	Patient	G6	66	4.21	Caucasian	yes	yes		Sample 19
Case89-SEQ 3	Patient	G7	66	3.5	Caucasian	yes	yes		Sample 32
Case8-SEQ 3	Patient	G6	73	4.4	African American	yes	yes		Sample 17
Z11B-SEQ 3	Control	Control	24	--	Caucasian	yes	yes		Sample 2
Z12B-SEQ 3	Control	Control	23	--	Caucasian	yes	yes	yes	Sample 1

**Table A2:** Raw *C*q values of exploratory qRT-PCR analysis of blood samples from controls (*n* = 8) and patients (*n* = 8) to identify normalizer microRNAs for confirmatory qRT-PCR analysis. *C*q values are the average of triplicates (SD ≤ 0.2).

Sample	Sample Type	miR197-3p	miR26b-5p	miR296-5p	miR23a-3p	miR146a-5p	miR 1248	miR1468-3p	miR1538
7	Control	22.74829	21.628092	26.089388	18.800253	27.064646	19.802492	30.705496	31.02948
10	Control	21.136293	25.414007	32.061626	18.889477	26.500956	16.915016	30.051926	29.690506
1	Control	22.46137	19.360636	24.926224	17.68174	25.192787	17.260515	29.683975	30.3445345
8	Control	29.555853	27.222218	31.271189	19.254599	29.127176	21.902388	33.016678	32.38471
5	Control	20.826368	23.358286	28.328314	19.247988	26.861567	16.149588	26.136414	28.535395
9	Control	22.516693	23.018911	27.688248	21.737234	28.745064	18.812151	29.16825	29.95835
3	Control	22.004297	21.192694	26.759714	17.74209	25.819382	15.312261	26.210001	29.330034
4	Control	20.42111	21.1323	27.237703	29.127176	26.609123	16.747427	26.76587	28.660437
22	Patient	21.886065	23.961235	30.295868	17.754295	26.563072	16.856455	28.191488	29.235413
14	Patient	28.050713	27.764153	32.219086	21.700003	28.97425	22.934767	33.360752	32.80031
23	Patient	20.232058	20.535894	28.087671	15.586144	25.002638	20.58262	27.846542	28.899343
24	Patient	20.934057	20.589144	26.781439	16.453804	24.992987	15.296409	28.57466	29.60646
15	Patient	20.324524	21.27265	28.345781	23.688972	31.2935	16.856455	31.182531	32.48879
25	Patient	22.465195	22.984718	26.560272	21.267275	27.856596	20.140722	28.645343	30.621424
16	Patient	21.8991	23.907438	28.562735	21.182196	27.760199	18.519064	27.570486	30.55234
33	Patient	19.746403	22.80751	28.66214	18.871817	27.803125	17.341208	27.578966	28.894281

**Table A3:** Raw *C*q values of confirmatory qRT-PCR analysis of blood samples from controls (*n* = 10) and patients (*n* = 26). *C*q values are the average of triplicates (SD ≤ 0.2).

Number	Sample Type	miR127-3p	miR1468-3p	miR146a-5p	miR1538	miR197-3p	miR204-5p	miR20a-5p	miR32-5p	miR329-3p	miR454-3p	miR487b-3p
1	Control	36.908432	30.543045	25.765848	32.149563	20.931528	30.270754	17.96527	25.546413	36.75606	22.844046	36.028534
2	Control	37.981623	28.062347	24.830622	30.136032	19.910437	36.117193	15.91111	23.228275	32.757341	22.673447	34.425587
3	Control	28.144186	28.446253	25.333971	30.515898	20.662188	30.312696	18.96527	29.557442	29.08623	25.073465	31.28196
4	Control	28.527613	29.671013	26.744165	30.476606	20.475235	30.221922	19.232796	29.752726	29.638098	26.315996	32.190907
5	Control	30.057663	29.518211	26.824263	28.535395	20.943573	30.793854	22.753603	26.935396	30.549356	28.72201	33.296204
6	Control	30.720682	29.358604	24.906527	31.398394	21.780424	29.43325	20.239492	30.17968	29.987643	25.529074	33.19684
7	Control	30.600016	31.048105	27.055967	33.107254	21.905685	31.476599	20.894003	27.493944	30.090225	24.6573	32.67403
8	Control	33.510212	30.512245	28.752623	29.60646	21.71274	30.86007	19.256899	31.885645	29.467866	23.78831	30.392588
9	Control	29.977875	29.733969	27.0654	29.95835	23.657553	30.979445	20.109362	27.893682	32.036114	25.58172	29.871424
10	Control	28.420685	30.848099	26.421408	32.04854	20.955032	30.269693	25.617834	27.432236	29.530313	29.78074	33.72329
11	Patient	25.514578	28.478271	23.705612	30.452873	22.588583	27.87504	23.345776	28.470451	23.393488	25.4003	25.936775
12	Patient	28.76194	28.892448	24.489096	31.246782	21.041815	29.743967	23.559605	27.84227	26.779068	27.28158	29.112656
13	Patient	28.005022	28.854507	23.039087	32.001001	21.060934	28.308046	23.194078	26.389101	27.779022	27.499535	29.67564
14	Patient	23.12572	30.165638	25.97425	34.46028	20.858582	23.945423	24.633371	33.6321	24.79482	28.784933	31.840466
15	Patient	25.970835	28.054586	22.90155	32.48879	26.455523	27.135048	25.562134	35.949183	27.743675	23.302767	29.316404
16	Patient	30.06621	31.15792	24.450277	31.151573	21.628485	23.59674	20.898695	32.535934	26.933802	28.414148	23.654408
17	Patient	29.233658	29.118433	24.842314	31.093117	22.109777	29.91673	28.335602	27.745428	29.466345	24.386953	30.998627
18	Patient	24.328703	28.683714	29.701113	30.66745	19.658997	25.6975	29.903137	30.435247	25.765816	26.52373	29.824835
19	Patient	21.014902	29.878317	31.95202	32.125797	17.540724	25.09019	26.061022	34.43073	22.8402	32.833908	26.911285
20	Patient	26.311941	28.65756	22.998682	32.747137	25.313272	26.886536	26.856186	27.684212	24.394753	33.664633	20.06597
21	Patient	26.961014	26.03805	22.823648	28.527018	27.915817	25.870775	30.0119	29.701286	26.257238	25.049133	20.115797
22	Patient	27.535292	28.895426	26.545149	30.66663	20.694304	29.86962	24.83243	35.122456	27.294931	29.74388	21.432922
23	Patient	29.402014	29.05626	25.683632	28.899343	20.198837	28.838037	22.598902	32.26222	28.878447	27.629982	22.840466
24	Patient	26.196487	29.45417	24.96972	32.38471	20.566633	29.10848	20.786528	28.78074	29.102547	35.674986	22.73802
25	Patient	29.829214	29.245398	25.854036	30.621424	20.477509	27.870811	29.884722	27.046827	20.211077	34.532679	23.396152
26	Patient	29.333616	29.284616	25.595312	30.323542	20.69581	29.889395	29.074312	27.739935	28.65606	34.9503	31.2132
27	Patient	26.915873	28.553482	24.359283	29.934687	23.699072	28.018053	28.724783	29.167513	25.274988	35.780039	27.273096
28	Patient	29.36677	29.126135	25.857595	31.09918	20.901865	27.674309	29.272202	27.905403	25.156372	34.727425	30.325487
29	Patient	30.076767	27.860743	24.468689	32.051258	21.122679	27.960495	20.559242	31.840103	25.185661	25.716452	21.408869
30	Patient	29.482193	28.605219	24.295252	30.460472	21.434538	21.330612	29.717953	29.96257	25.31598	25.70342	21.854279
31	Patient	29.213333	29.811007	26.022947	30.650206	20.445127	27.530617	21.284222	31.529474	25.304756	28.699488	30.818052
32	Patient	27.923819	28.06331	22.805868	29.62851	25.487047	30.44813	28.31951	27.352371	25.182838	34.379427	29.983652
33	Patient	29.104956	30.537256	28.080553	31.083038	20.618134	27.849699	21.825888	33.01379	20.256021	27.8463	23.03477
34	Patient	29.28003	28.26641	24.822348	30.753214	19.842566	26.921324	21.533236	30.028503	26.876923	35.232162	25.622961
35	Patient	28.177826	28.581133	23.973612	30.277636	20.03753	27.943693	31.369062	35.510693	26.46978	28.047266	20.259644
36	Patient	20.407839	28.817247	26.935812	30.680155	21.042847	31.687258	33.005196	32.28391	19.864021	28.640589	23.454853

**Table A4:** Normalized *C*q values for confirmatory qRT-PCR analysis of blood samples from controls (*n* = 10) and patients (*n* = 26). *C*q values are the average of triplicates (SD ≤ 0.2).

Number	Sample Type	miR127-3p	miR204-5p	miR20a-5p	miR32-5p	miR329-3p	miR454-3p	miR487b-3p
1	Control	9.932081552	3.294403552	−9.011080448	−1.429937448	9.779709552	−4.132304448	9.052183552
2	Control	12.55318916	10.68875916	−9.51732384	−2.20015884	7.32890716	−2.75498684	8.99715316
3	Control	2.181017005	4.349527005	−6.997898995	3.594273005	3.123061005	−0.889703995	5.318791005
4	Control	2.000546379	3.694855379	−7.294270621	3.225659379	3.111031379	−0.211070621	5.663840379
5	Control	3.829786882	4.565977882	−3.474273118	0.707519882	4.321479882	2.494133882	7.068327882
6	Control	4.128401402	2.840969402	−6.352788598	3.587399402	3.395362402	−1.063206598	6.604559402
7	Control	2.662065759	3.538648759	−7.043947241	-0.444006241	2.152274759	−3.280650241	4.736079759
8	Control	6.106260714	3.456118714	−8.147052286	4.481693714	2.063914714	−3.615641286	2.988636714
9	Control	2.496480815	3.498050815	−7.372032185	0.412287815	4.554719815	−1.899674185	2.390029815
10	Control	1.220601618	3.069609618	−1.582249382	0.232152618	2.330229618	2.580656618	6.523206618
11	Patient	−0.590230547	1.770231453	−2.759032547	2.365642453	−2.711320547	−0.704508547	−0.168033547
12	Patient	2.645655116	3.627682116	−2.556679884	1.725985116	0.662783116	1.165295116	2.996371116
13	Patient	2.133020459	2.436044459	−2.677923541	0.517099459	1.907020459	1.627533459	3.803638459
14	Patient	−4.268868334	−3.449165334	−2.761217334	6.237511666	−2.599768334	1.390344666	4.445877666
15	Patient	−1.289403891	−0.125190891	−1.698104891	8.688944109	0.483436109	−3.957471891	2.056165109
16	Patient	3.299833617	−3.169636383	−5.867681383	5.769557617	0.167425617	1.647771617	−3.111968383
17	Patient	2.67829958	3.36137158	1.78024358	1.19006958	2.91098658	−2.16840542	4.44326858
18	Patient	−2.442123688	−1.073326688	3.132310312	3.664420312	−1.005010688	−0.247096688	3.054008312
19	Patient	−6.067611542	−1.992323542	−1.021491542	7.348216458	−4.242313542	5.751394458	−0.171228542
20	Patient	−0.875350601	−0.300755601	−0.331105601	0.496920399	−2.792538601	6.477341399	−7.121321601
21	Patient	0.732417073	−0.357821927	3.783303073	3.472689073	0.028641073	−1.179463927	−6.112799927
22	Patient	1.121376344	3.455704344	−1.581485656	8.708540344	0.881015344	3.329964344	−4.980993656
23	Patient	3.711214401	3.147237401	−3.091897599	6.571420401	3.187647401	1.939182401	−2.850333599
24	Patient	−0.259009595	2.652983405	−5.668968595	2.325243405	2.647050405	9.219489405	−3.717476595
25	Patient	3.588714461	1.630311461	3.644222461	0.806327461	−6.029422539	8.292179461	−2.844347539
26	Patient	3.144817167	3.700596167	2.885513167	1.551136167	2.467261167	8.761501167	5.024401167
27	Patient	0.412117905	1.514297905	2.221027905	2.663757905	−1.228767095	9.276283905	0.769340905
28	Patient	2.915212989	1.222751989	2.820644989	1.453845989	−1.295185011	8.275867989	3.873929989
29	Patient	4.012268775	1.895996775	−5.505256225	5.775604775	−0.878837225	−0.348046225	−4.655629225
30	Patient	3.528189418	−4.623391582	3.763949418	4.008566418	−0.638023582	−0.250583582	−4.099724582
31	Patient	2.808155968	1.125439968	−5.120955032	5.124296968	−1.100421032	2.294310968	4.412874968
32	Patient	1.55724467	4.08155567	1.95293567	0.98579667	−1.18373633	8.01285267	3.61707767
33	Patient	1.877809959	0.622552959	−5.401258041	5.786643959	−6.971125041	0.619153959	−4.192376041
34	Patient	3.700034457	1.341328457	−4.046759543	4.448507457	1.296927457	9.652166457	0.042965457
35	Patient	2.785951344	2.551818344	5.977187344	10.11881834	1.077905344	2.655391344	−5.132230656
36	Patient	−6.198563209	5.080855791	6.398793791	5.677507791	−6.742381209	2.034186791	−3.151549209

**Table A5:** Comparison of fold change in blood HTS data to tissue HTS data from the TCGA database. Fold change was calculated in the same method as panel members using the Edge R program.

MicroRNA	Fold Change in Blood *	Fold Change in TCGA **
hsa-miR-32-5p	−4.11	1.71
hsa-miR-329-3p	4.29	−1.27
hsa-miR-487b-3p	6.06	−1.66
hsa-miR-454-3p	−1.91	1.26
hsa-miR-204-5p	3.43	−3.81
hsa-miR-20a-5p	−2.11	2.19
hsa-miR-127-3p	2.69	−1.43

* FDR < 0.2; ** FDR < 0.05.

**Table A6:** Chromosome 14 q32.31 dysregulated microRNAs in our analysis of blood samples compared to data from the TCGA tissue database. In addition to our proposed panel members (miR-329-3p, miR-487b-3p and miR-127-3p), subsequent analysis of HTS data uncovered five other microRNAs from this locus (miR-654-5p, miR-654-3p, miR-493-3p, miR-493-5p and miR-433-3p) to be upregulated in blood. Since these microRNAs were not within the top ten potential candidates, they were not carried forth for qRT-PCR confirmation. However, analysis of the TCGA database did confirm these microRNAs to be tumor suppressors lost in tumor tissue compared to matched tumor-free margins, fitting with our model that tumors may strive to get rid of tumor suppressors in order to progress. Fold change is shown in a Log2 scale and was calculated with the same method as panel members using the edge R program.

miRNA	Dysregulation Ranking in Blood	Log Fold Change in Blood	Log Fold Change in TCGA
miR-329-3p	4	2.10	−0.34
miR-487b-3p	5	2.60	−0.73
miR-127-3p	9	1.43	−0.52
miR-654-5p	17	1.96	−0.61
miR-654-3p	36	1.08	−0.61
miR-493-3p	37	1.74	−0.58
miR-493-5p	59	1.29	−0.58
miR-433-3p	43	1.30	−0.16
